# Editorial: Sleep and cognition: the role of sleep patterns and deprivation in memory and learning

**DOI:** 10.3389/fnbeh.2026.1981387

**Published:** 2026-10-20

**Authors:** Simone Marie Ota, Robbert Havekes, Paula Ayako Tiba

**Affiliations:** 1 Centre for Neural Circuits and Behaviour, University of Oxford, Oxford, United Kingdom; 2 Groningen Institute for Evolutionary Life Sciences (GELIFES), University of Groningen, Groningen, Netherlands; 3 Center for Mathematics, Computing and Cognition (CMCC), Universidade Federal do ABC (UFABC), São Bernardo do Campo, Brazil

**Keywords:** cognitive performance, memory consolidation, sleep architecture, sleep quality, slow-wave sleep

This Special Research Topic was conceptualized to bridge the gap between basic neurophysiological mechanisms of sleep and their real-world cognitive implications across the lifespan. While the essential roles of slow-wave sleep (SWS) microstructures and rapid eye movement (REM) sleep in memory consolidation are well-established, how these neural processes interact with daily lifestyle stressors, shift work, cultural demands, and aging remains incompletely understood. The primary objective of this Research Topic was to bring together multi-level evidence—spanning high-density EEG, event-related potentials, wearable in-home monitoring, and large-scale epidemiological cohorts—to elucidate how sleep patterns, deprivation, and night-to-night variability shape cognitive integrity in diverse populations.

Sleep significantly contributes to memory consolidation and cognitive performance. In particular, oscillatory events occurring during SWS, such as slow oscillations (SOs) and sleep spindles, are crucial for reorganizing and transferring new information acquired during wakefulness, from the hippocampus to the neocortex, while REM sleep plays a vital role in emotional memory processing (Wilson and McNaughton, 1994; Marshall and Born, 2007; Diekelmann and Born, 2010). Therefore, it is not surprising that sleep deprivation has negative consequences on memory consolidation (Crowley et al., 2024). Moreover, once sleep architecture changes across lifespan, with progressive difficulty in sleep maintenance and percentage of SWS decreasing with age (Ohayon et al., 2004), it is essential to study the influence of sleep in cognition in different age groups. The articles compiled in this Research Topic investigate these neurophysiological underpinnings, examine the impacts of acute and chronic sleep deprivation, and elucidate how sleep quality and night-to-night variability affect cognitive function across diverse populations, from young students to older adults.

Exploring the microstructural mechanisms of sleep, Keeble et al. present a mini-review detailing how the precise temporal coordination between SOs, sleep spindles, and hippocampal ripples during SWS facilitates memory transfer and synaptic plasticity. They discuss how SWS disruptions—driven by aging, neurological disorders, and pharmacotherapy—lead to memory impairments, and highlight the potential of emerging neuromodulation techniques (e.g., closed-loop acoustic stimulation) to restore sleep microstructure and enhance cognitive health.

Taking a behavioral approach to sleep-dependent memory triage, Kurdziel et al. investigate how intentional top-down goals interact with emotional salience during overnight sleep, since it is known that executive control processes can suppress certain memories to be recalled (Anderson and Green, 2001). They applied a task with remember vs. forget instructions of neutral and negative words in sleep or wake condition, and demonstrated that percentage of SWS and delta power were negatively associated with total recall, while sleep spindle correlated with negative remember-cued words. Besides, REM theta power was associated with negative foils intrusions. The study shows that top-down instruction overrides emotional salience when the cues are competing and sleep events associated with selective memory consolidation.

The critical role of sleep quality in the cognitive outcomes of younger cohorts is further highlighted in two student-focused studies. Xu et al. demonstrate a high prevalence of insomnia (54.1%) and poor sleep quality (58.9%) among college students in Guangzhou. They show that short sleep duration exacerbates sleep disturbances, which directly correlate with declines in attention, language, and visual-perceptual learning, placing students at risk of long-term cognitive decline. This vulnerability is modulated by sociocultural contexts, as demonstrated by Ampofo et al. in a cross-cultural study comparing university students in Tokyo and London. They find that Tokyo students suffer from significantly poorer sleep quality, had shorter sleep duration and lower sleep efficiency compared to their London peers, and that cultural factors—such as intense academic pressure and societal norms prioritizing productivity over rest in Japan—significantly moderate the relationship between sleep quality and memory, making the detrimental impact of poor sleep on verbal learning more pronounced in Tokyo. This study suggests that interventions to improve sleep quality and cognitive functioning in university students need to consider cultural and lifestyle differences.

When sleep is severely disrupted or altogether absent, the cognitive consequences are immediate. Ren et al. investigate the impacts of 24-h total sleep deprivation (TSD) on healthy adults, combining subjective reaction times (using the auditory Oddball paradigm) with objective auditory P300 event-related potentials (ERPs). They show that acute sleep loss significantly extends P300 latency, indicating delayed auditory cognitive processing and reduced attentional alertness. Interestingly, they compare individuals with and without a chronic history of night-shift work (>24 months). While acute sleep loss dramatically impairs reaction times in the non-shift group, chronic night-shift workers show persistent baseline alterations in cognitive metrics, suggesting that chronic sleep disruptions induce cumulative, unrestored neurobiological damage.

Addressing the challenge of capturing naturalistic sleep-cognition relationships, Brooks et al. present a multi-night, in-home wearable EEG study in healthy older adults. They showed that even with participants putting the EEG device themselves, ~71% of the recorded data was usable. Over approximately 10 nights, they demonstrate substantial night-to-night variability in both macro- and micro-architectural sleep metrics (e.g., WASO, SO, and spindle density). Interestingly, despite clear nightly fluctuations, they find that next-day executive functions (inhibition and cognitive flexibility) remain resilient to daily sleep variations.

As the global population ages, understanding the relationship between sleep patterns and cognitive decline becomes paramount. In a large-scale, 9-year longitudinal study of 9,970 older Chinese adults, Qian et al. explore the links between sleep patterns and cognitive frailty. Older Chinese adults with poor sleep quality and short sleep duration (< 6 h) had 67% and 48% higher risk of cognitive weakness, respectively, while no and excessive napping, increased the risk of cognitive debilitation by 23% and 69%, respectively. This and Brooks' study underscores the feasibility of using portable devices to conduct ecologically valid sleep research, while providing crucial statistical parameters (variance components and effect sizes) to guide the design of future longitudinal studies in different populations.

Together, the contributions to this Research Topic span the full spectrum of sleep and cognitive science. They transition from laboratory-based microstructural investigations and acute deprivation experiments to naturalistic, multi-night home monitoring and large-scale demographic epidemiological cohorts. By integrating these multi-level methodologies, this Research Topic moves the field forward beyond traditional single-night paradigms toward ecologically valid and lifespan-oriented sleep science. Nevertheless, key open questions remain: What neural compensatory mechanisms allow certain cognitive functions to remain resilient despite night-to-night sleep fluctuations, and how can micro-architectural sleep biomarkers be leveraged for the early detection of cognitive frailty? Collectively, these studies reinforce the profound, multi-dimensional impact of sleep on brain health, and highlight the urgent need for more research in age and culturally diverse populations, and in pharmacological or behavioral approaches to prevent or mitigate the cognitive impacts of sleep deprivation. Those interventions should be culturally tailored, occupational, and age-specific to preserve cognitive integrity across the lifespan.
